# Exploring the impact of *Helicobacter pylori* on gut microbiome composition

**DOI:** 10.1371/journal.pone.0218274

**Published:** 2019-06-18

**Authors:** Nihar Ranjan Dash, Ghalia Khoder, Aml Mohamed Nada, Mohammad Tahseen Al Bataineh

**Affiliations:** 1 College of Medicine, University of Sharjah, Sharjah, United Arab Emirates; 2 College of Pharmacy, University of Sharjah, Sharjah, United Arab Emirates; 3 University Hospital Sharjah, Sharjah, United Arab Emirates; 4 Research Institute for Medical & Health Sciences at University of Sharjah, Sharjah, United Arab Emirates; Oita University Faculty of Medicine, JAPAN

## Abstract

*Helicobacter pylori* (*H*. *pylori*) is known to colonize gastric mucosa, induce inflammation, and alter gastric microbiota resulting in a spectrum of gastric diseases. Likewise, changes in gut microbiota have recently been linked with various metabolic and inflammatory diseases. While extensive number of studies were published examining the relationship between *H*. *pylori* and gastric microbiota, little is known about the impact of *H*. *pylori* on downstream gut microbiota. In this study, we performed 16 S rRNA and ITS2-based microbial profiling analysis of 60 stool samples from adult individuals. Remarkably, the gut microbiota of *H*. *pylori* infected individuals was shown to be increased of members belonging to Succinivibrio, Coriobacteriaceae, Enterococcaceae, and Rikenellaceae. Moreover, gut microbiota of *H*. *pylori* infected individuals was shown to have increased abundance of *Candida glabrata* and other unclassified Fungi. These results links possible role for *H*. *pylori*-associated changes in the gut microbiota in intestinal mucosal barrier disruption and early stage colorectal carcinoma deployment. Altogether, the identified differences in bacterial and fungal composition provides important information that may eventually lead to the development of novel biomarkers and more effective management strategies.

## Introduction

*Helicobacter pylori* (*H*. *pylori*) is a gram-negative microaerophilic bacterium that colonizes the gastric mucosa of more than half of the worldwide population with high geographic variability [1]. *H*. *pylori* infection is generally acquired during childhood and can persist life-long without symptoms. It triggers pathogenesis by creating reactive oxygen species and modulating host-inflammatory responses. This pathogen is known to cause diseases of the upper gastrointestinal tract such as peptic ulcer, gastric cancer, and gastric mucosa-associated lymphoid tissue (MALT) lymphoma [2, 3]. Further, recent studies have linked *H*. *pylori* infection to lower gastrointestinal tract diseases, such as colorectal cancer [4, 5]. Several factors affects the outcome of *H*. *pylori* infection, including virulence properties such as sialic acid-binding adhesin (SabA), vacuolating cytotoxin (VacA), and cytotoxin-associated gene A (CagA) [6, 7]. In addition, host genetic, immunogenic factors, and environmental factors including resident gut microbiota is known to play a significant role in disease pathogenesis [8].

Studies have suggested that gut microbiota can be affected by *H*. *pylori* infection [8, 9]. In fact, infection with *H*. *pylori* associates with altered gastric microbiota and dysbiosis that has been implicated in the pathogenesis of gastric diseases [10, 11]. However, there is a lack of clarity whether *H*. *pylori* infection itself supports the growing of undesirable microorganisms or inversely, an altered microbiota creates advantageous conditions for *H*. *pylori* colonization. It is very likely that a multifaceted interaction exists, in which the colonizing *H*. *pylori* favors the growing of certain microbes and *vice versa*. Perhaps dysbiosis promote changes in gastric mucosa that is more favorable for *H*. *pylori* colonization [8].

Currently, the exact interaction between *H*. *pylori* and gut microbiota is not fully understood and reported literature shows contradictory results. For example, a study in children reported that the gut microbiota of *H*. *pylori*-negative patient presented more relative abundance of gammaproteobacteria, betaproteobacteria, bacteroidia and clostridia classes and a higher bacterial richness and diversity [12]. Whereas, another study reported a contrasting result that children infected with *H*. *pylori* presented increased number of gut microbiota including *Proteobacteria*, *Clostridium*, *Firmicutes* and *Prevotella* in comparison to patients without the infection [13]. Whether these differences are of primary etiological importance to *H*. *pylori* infection or secondary to the altered inflammatory and metabolic environments remains largely unknown. Aforementioned deliberations have necessarily created growing interest exploring the potential interactions between the human gut microbiota and *H*. *pylori*. In this study, we aim explore and characterize the gut microbiome composition between asymptomatic *H*. *pylori* infected *versus* non-infected subjects to better understand the interplay between *H*. *pylori* and gut microbiota and its effect on human health and disease conditions.

## Material and methods

### Ethical statement

The study was performed after receiving the necessary ethical approval University Hospital Sharjah- Hospital Ethics Research Committee (UHS-HERC-021-07022017). All subjects were recruited at University Hospital Sharjah (Sharjah, UAE) and provided written informed consent.

#### Stool Sample collection and preparation

We collected 60 stool specimens from Emirati citizens adults. The basic demographic information such as age, gender, marital status, education level, diet, exercise, height and weight were documented. From each subject, 2 to 4 gram of freshly passed stool specimen was collected in a sterile container stored immediately into liquid nitrogen and then transferred to −80°C for further analysis. Liquid (diarrheal) stool and use of antibiotics in the last 3 months or use of proton pump inhibitors and/or bismuth preparations among the volunteers were the only exclusion criteria used in this study.

### *H*. *pylori* detection

Antigen detection was performed on all 60 stool samples using Premier Platinum HpSA immunoassay (Meridian Bioscience Inc.). Tests were performed in duplicate according to the manufacturer`s instructions. Spectrophotometric absorbance at dual wavelength 450/630 nm equal or above 0.1 was considered a positive result.

### DNA extraction

Faecal samples were subjected to DNA extraction using QIAampPowerFecalDNA kit (Qiagen Ltd. GmbH, Germany) following the manufacturer’s instruction (Qiagen Ltd.). The extracted DNA was stored at −80°C for further analysis.

### PCR, sequencing, and sequence processing

Bacterial 16S rRNA genes were amplified using polymerase chain reaction (PCR) targeting the V4 region with dual-barcoded, as per the procedure of Kozich et al. (2013) [14]. Next, amplicons were sequenced with an Illumina MiSeq using the 250-bp paired-end kit (v.2). Sequences were denoised, taxonomically classified using Greengenes (v. 13_8) as the reference database, and clustered into 97%-similarity operational taxonomic units (OTUs) with the mothur software package (v. 1.39.5) (Schloss et al. 2009) [15], following the recommended procedure (https://www.mothur.org/wiki/MiSeq_SOP; accessed Nov 2017). ITS2 region were sequenced on an Illumina MiSeq (v. 2 chemistry) using the dual barcoding protocol of Kozich et al. (2014). Primers and PCR conditions used for 16S sequencing are identical to those of Kozich et al.; those used for ITS2 sequencing were described by Gweon et al. (2015) [16]. Bacterial sequences were processed and clustered into operational taxonomic units (OTUs) with the mothur software package (v. 1.39.5) (Schloss et al. 2009), following the recommended procedure (https://www.mothur.org/wiki/MiSeq_SOP; accessed Aug 2018). Paired-end reads were merged and curated to reduce sequencing error (Huse et al. 2010). The processing pipeline was identical as the one used for bacteria, except for the following differences: (1) paired-end reads were trimmed at the non-overlapping ends, and (2) high quality reads were classified using UNITE (v. 7.1) (Kõljalg et al., 2005) as the reference database. A consensus taxonomy for each OTU was obtained and the OTU abundances were then aggregated into genera. OTU abundances were summarized with the Bray-Curtis index and a principle coordinate analysis (PCoA) was performed to visualize microbiome similarities and a permutational analysis of variance (PERMANOVA) was done to test the significance of groups. Alpha diversity was calculated using Shannon’s diversity index.

### Quality control

The possibility for contamination was examined by co-sequencing DNA amplified from samples and from four each of template-free controls and extraction kit reagents treated the same way as the samples. Two positive controls, consisting of cloned SUP05 DNA, were also added (number of copies = 2*10^6). Operational taxonomic units were considered putative contaminants (and were removed) if their mean abundance in controls reached or surpassed 25% of their mean abundance in samples.

### Statistical analysis

Alpha diversity was assessed with the Shannon index on raw OTU abundance tables after filtering out contaminants. The significance of diversity changes was tested with an ANOVA. To evaluate beta diversity across samples, we excluded OTUs occurring in fewer than 10% of the samples with a count of less than three and calculated Bray-Curtis indices. We tested beta diversity, underscoring differences across samples, using principal coordinate analysis (PCoA) ordination. Dissimilarity in community structure was assessed with permutational multivariate analyses of variance (PERMANOVA) with treatment group as the main fixed factor and using 4,999 permutations for significance testing. All analyses were conducted in the R environment.

## Results and discussion

### 1. Bacterial and fungal sequence curation analysis and taxonomic composition

Stool samples from the 60 individuals enrolled in this study were obtained in order to assess the microbiota composition and *H*. *pylori* infection. From these 60 individuals, 12 were *H*. *pylori* infected by antigen detection test and their clinical characteristics shown in S1 Table. We sequenced 16S rRNA gene V4 amplicons generated from DNA samples on a MiSeq. MiSeq-generated Fastq files were quality-filtered and clustered into 97% similarity operational taxonomic units (OTUs) using the mothur software package [http://www.mothur.org]. The resulting dataset had 21257 OTUs (including those occurring once with a count of 1, or singletons). An average of 18383 quality-filtered reads were generated per sample. Sequencing quality for R1 and R2 was determined using FastQC 0.11.5, and visualized (S1 Fig). We sequenced ITS2 amplicons generated from DNA samples on a MiSeq. MiSeq-generated Fastq files were quality-filtered and clustered into 97% similarity operational taxonomic units (OTUs) using the mothur software package [http://www.mothur.org]. The resulting dataset had 3171 OTUs (including those occurring once with a count of 1, or singletons). An average of 9581 quality-filtered reads were generated per sample. Sequencing quality for R1 and R2 was determined using FastQC 0.11.5, and visualized (S2 Fig).

Examination of the taxonomic composition generated from high quality reads and classified using Greengenes v. 13_8 as the reference database. We aggregated OTUs into each taxonomic rank, and plotted the relative abundance of the most abundant ones. In the figure legends, the unfilled portion of the bar represents unclassified and lower-abundance taxa (S3 and S4 Figs).

### 2. Gut microbiota of *H*. *pylori* infected subjects show higher level of complexity compared to uninfected subjects

**Fig 1:**
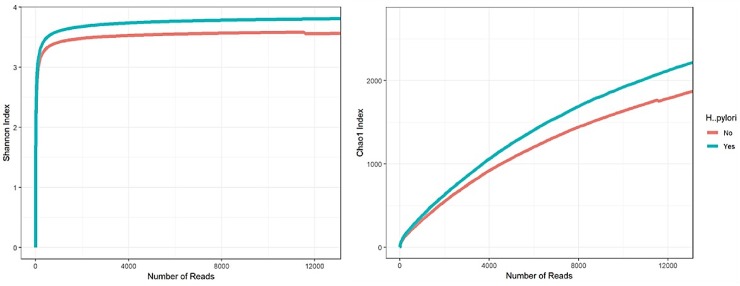
Gut microbiota of *H*. *pylori* infected subjects show higher level of complexity. Evaluation of the alpha-diversity in the 60 analyzed samples. Outlined reports the average rarefaction curves based on the Chao1 and Shannon index at increasing sequencing depth of *H*. *pylori* infected and healthy samples. *H*. *pylori* infected and healthy datasets are colored in blue and red, respectively.

To evaluate the intra- and inter-individual variability among *H*. *pylori* infected and uninfected subjects, we first evaluated averaged alpha-diversity for the 60 samples, examination of the averaged rarefaction curves based on the biodiversity indices, Shannon and Chao1 at increasing sequencing depth exhibited that both curves incline to reach a plateau. Therefore, the generated sequences from all samples were considered adequate to cover most of the biodiversity in the samples. Surprisingly, and unlike what have been previously reported, average rarefaction curves for *H*. *pylori* infected and uninfected subjects revealed a difference in that, on average *H*. *pylori* infected samples show higher level of gut microbiota complexity compared to uninfected samples. Statistical analysis, calculated for the peak sub-sampling point reached by all samples, i.e. 18000 reads, showed that the two curves significantly vary based on a one-way analysis of variance (ANOVA) (p-value < 0.05) (Fig 1).

Numerous groups have demonstrated a significant effect of *H*. *pylori* on gastric microbial richness and relative abundance [17–20]. Suggesting a major role of gastric microbiota on *H*. *pylori*-induced gastric inflammation and cancer [19, 21]. However, little is known about the impact of *H*. *pylori* on downstream gut microbiota. Here, we report an intriguing difference in the gut microbiome between *H*. *pylori* infected and uninfected groups. Furthermore, It is known that obesity is associated with dysbiosis and decreased diversity [22–24]. In contrast, despite that 70% of the *H*. *pylori* infection subjects in our study were obese (BMI>30), here we show that *H*. *pylori* infected samples exhibits higher level of gut microbiota richness and complexity. This may suggest an important role for *H*. *pylori* in driving these compositional changes irrespective to BMI status.

*H*. *pylori* has been implicated in many uninfected, asymptomatic, and disease conditions [7, 8, 25]. Although biological attributes of higher microbial biodiversity have been evaluated favorably, especially against chronic inflammatory conditions such as obesity and inflammatory bowel diseases [25, 26]. Perhaps *H*. *pylori* presence induces changes in the gut micro-environmental cues, such as changes in pH that drives this compositional shift among native communities to compensate. Most likely this compensation will be translated into unique functional genes that are involved in important metabolic pathways.

**Fig 2:**
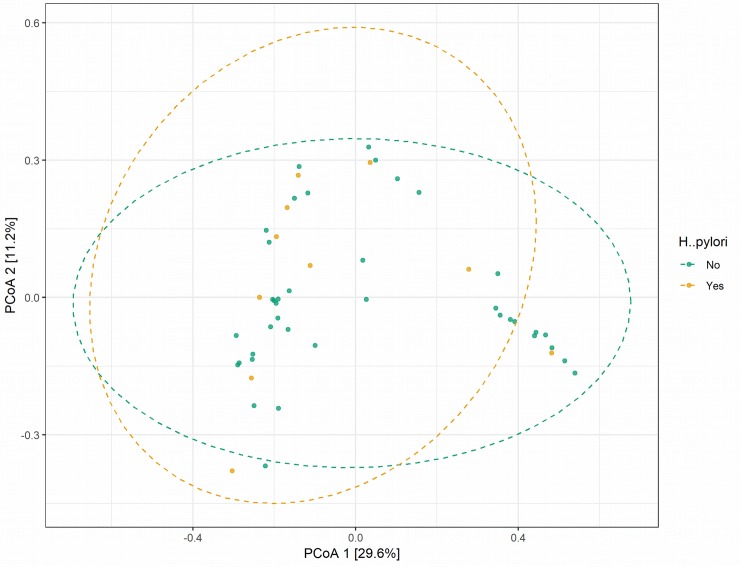
Evaluation of the beta-diversity in the 60 analyzed samples. Panel shows the predicted Principal Coordinate Analysis (PCoA). Healthy and *H*. *Pylori* data points and corresponding clusters are colored in green and orange respectively.

Next, we evaluated beta-diversity of microbiome composition, diversity among samples, we summarized OTU abundances into Bray-Curtis dissimilarities and performed a Principal Coordinate Analysis (PCoA). Based on permanova analysis no significant differences (p-value  = 0.9741) (Fig 2). Suggesting that *H pylori* infection only drives species richness (higher OTU count), but without affecting the overall inter- individual variability, the difference in microbiota composition between individuals. This observation is interesting to identify those microbial populations that are being more abundant as a result of *H*. *pylori* infection and to further understand their relevance in *H*. *pylori* pathogenesis and host immune interactions.

### 3. Exploration of bacterial abundance and prevalence between uninfected and *H*. *pylori* infected individuals

**Fig 3:**
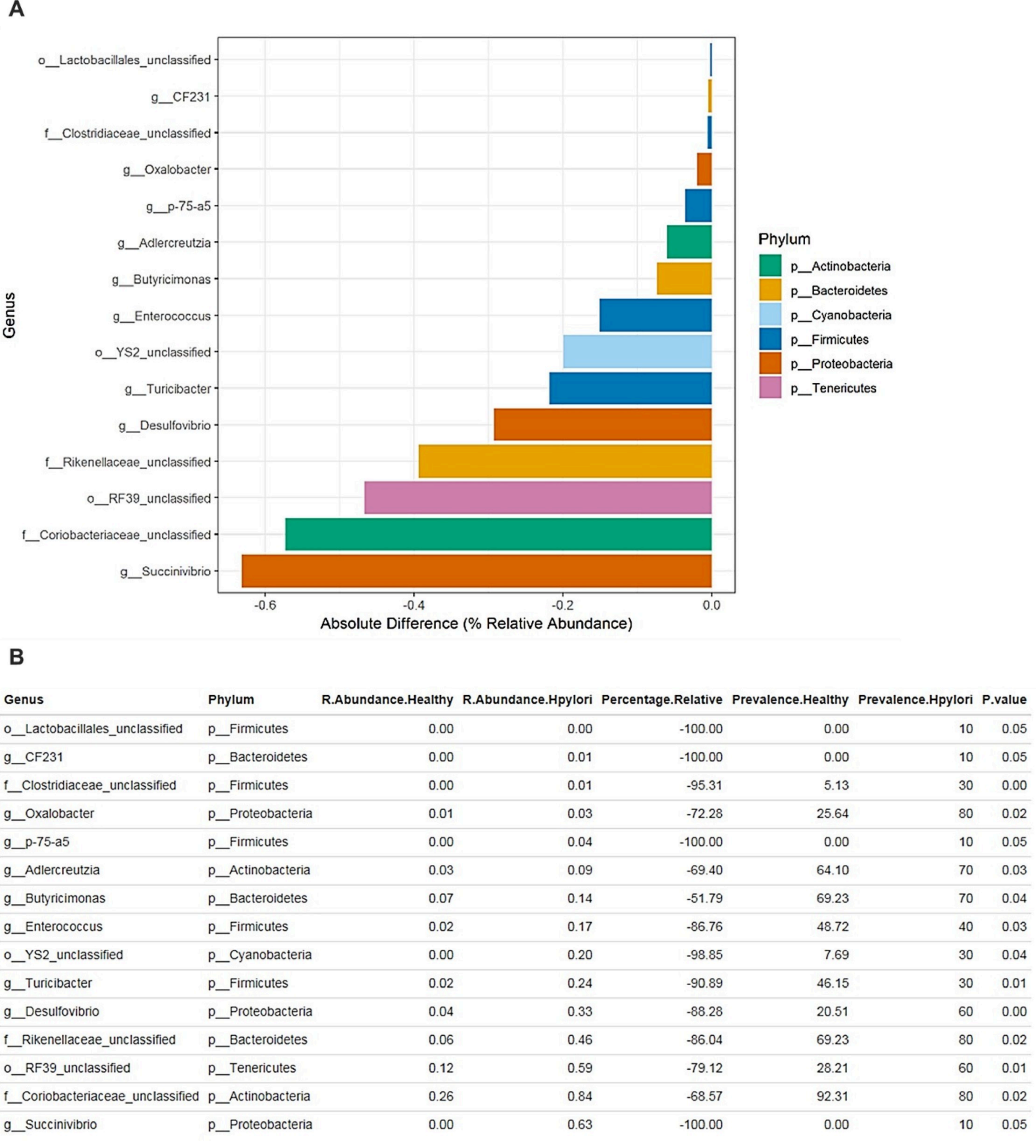
Exploration of the bacterial diversity in *H*. *pylori* infected and healthy subjects. (A)The bar plot reports only genera with an absolute percentage difference between *H*. *pylori* infected and healthy averages >0.1% and a p-value < 0.05, evaluated by means of ANOVA statistical analysis. (B) The table indicates the bacterial genera, the relative abundance and the prevalence of each group, the relative percentage difference and the p-value.

In order to evaluate possible differences in bacterial richness as suggested in Fig 1, we conducted ANOVA statistical analysis to compare the average relative abundance in uninfected and *H*. *pylori* infected groups of genera with an absolute percentage difference >0.1%. Remarkably, the comparison between uninfected and *H*. *pylori* infected datasets showed that profiles obtained from uninfected individuals have no statistically significant over-representation, however we characterized a statistically significant under-representation of Succinivibrio (% absolute −0.62%, p-value < 0.05), Coriobacteriaceae (% absolute −0.58%, p-value < 0.02), Enterococcaceae bacterium RF39 (% absolute −0.48%, p-value < 0.01) and Rikenellaceae (% absolute −0.499%, p-value < 0.02) among others (Fig 3).

The higher abundance in these genera in *H*. *pylori* infected individuals may be correlates *H*. *pylori* pathogenesis and early stage cancerous development. Previous studies have reported that iron deficiency is associated with the presence of *H*. *pylori* [27, 28], *that has been clearly* demonstrated in animal models as well [29]. Further, Iron deficiency accelerates Helicobacter pylori-induced carcinogenesis [30]. Here, we report that Succinivibrio and Turicibacter among others have increased relative abundance with *H*. *pylori* infected (Fig 3A) and have been reported else were to increase with iron depletion [31]. Furthermore, accumulating evidence links colorectal cancer development with microbiomes changes [32–35]. Here, we showed that Coriobacteriaceae among others have high abundance with *H*. *pylori* infected samples (Fig 3A). This interesting observation is consistent with previous studies that have reported Coriobacteriaceae tend to grow more in tumor niches and have been implicated as an early stage tumorigenic agent [34, 36]. Altogether, suggesting that *H*. *pylori* has a viable role in colorectal cancer development similar to what have been previously reported [37, 38]. Most likely, these data can be useful to provide screening biomarkers to predict early colorectal cancer development in individuals with *H*. *pylori* infection.

### 4. Exploration of fungal diversity between uninfected and *H*. *pylori* infected subjects

**Fig 4:**
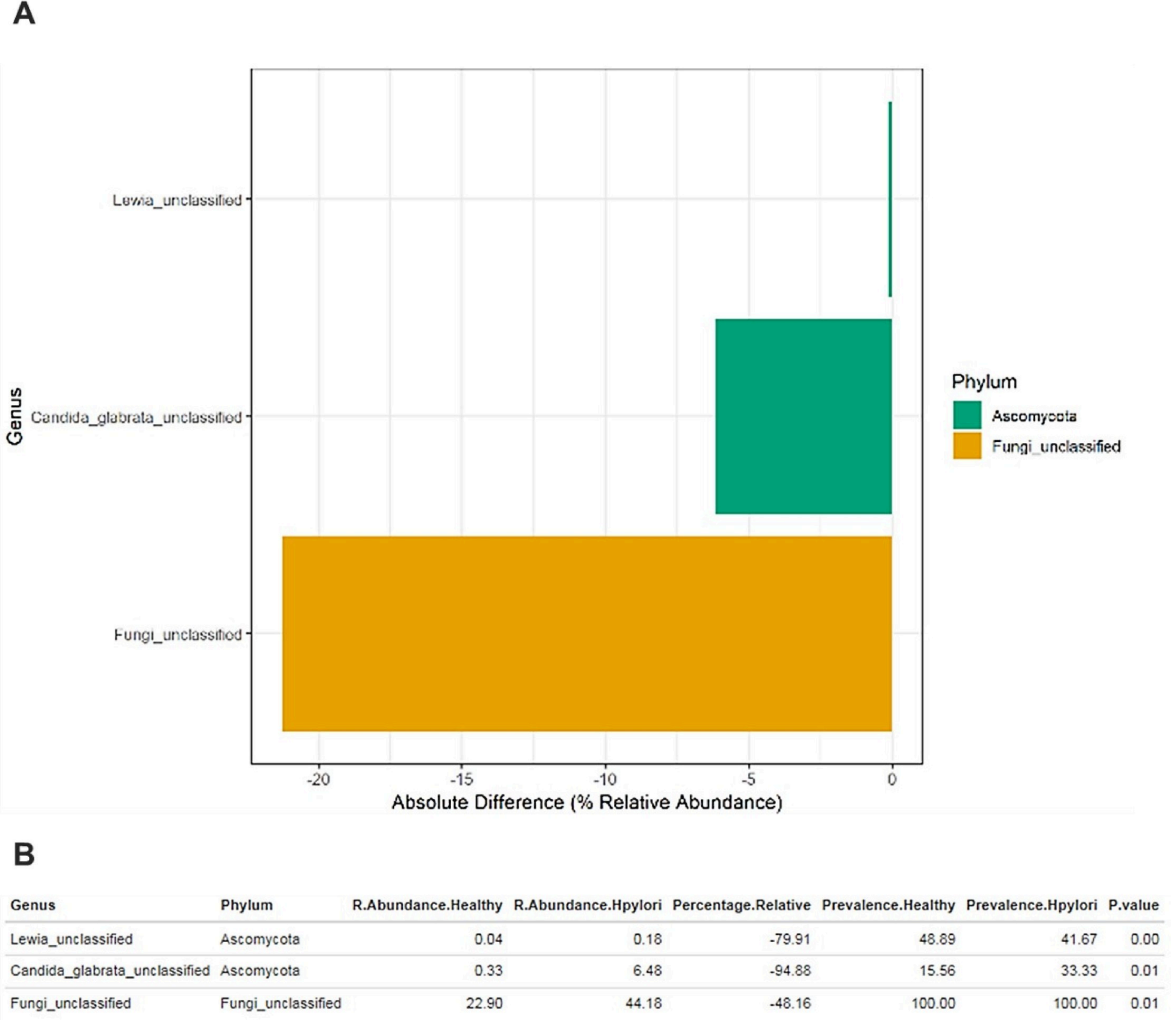
Exploration of the fungal diversity in *H*. *pylori* infected and healthy subjects. (A) The bar plot reports only genera with an absolute percentage difference between *H*. *pylori* infected and healthy averages >0.1% and a p-value < 0.05, evaluated by means of ANOVA statistical analysis. (B) The table indicates the fungal genera, the relative abundance and the prevalence of each group, the relative percentage difference and the p-value.

Mycobiota have been first characterized as members of the normal gut flora in 1967 [39], Fungal populations comprises a small percentage of the total gut microbiome. However, reports have indicated that these small numbers fungi, have surprisingly strong effects on dampening inflammatory responses in the gut [40, 41]. Also, others have reported their impact on bacterial community composition [42, 43]. In order to evaluate mycobiota in our study, we conducted ANOVA statistical analysis to compare the average relative abundance in uninfected and *H*. *pylori* infected groups of fungal genera with an absolute percentage difference >0.1%. The comparison between uninfected and *H*. *pylori* infected datasets showed that profiles obtained from uninfected individuals have no statistically significant over-representation, however we characterized a statistically significant under-representation of unclassified fungi (% absolute −21%, p-value < 0.01) and *Candida glabrata* (% absolute −6.3%, p-value < 0.01) among others (Fig 4).

In our study, Ascomycota is the most prevalent fungus phylum in both uninfected and *H*. *pylori* infected groups and similar to what have been published before [44]. However, we showed a staggering abundance of unclassified fungi (% absolute −21%, p-value < 0.01) in *H*. *pylori* infected *vs*. uninfected group. Suggesting a clear shift in the mycobiota as a result of *H*. *pylori* infection. Recent check (January, 2019) of the NCBI Genome database https://www.ncbi.nlm.nih.gov/genome/browse#!/eukaryotes/fungi revealed only 4029 complete fungal genomes compared with >182000 complete bacterial genomes. This suggest that fungi might be under-detected compared with bacteria in any sequencing efforts and underscores the importance of increasing the annotated reference sequences for fungi. Furthermore, fungi, as eukaryotes, are probably contributing in unique metabolic pathways to the microbial equilibrium and host interactions. This compositional shift from known commensal fungi to unclassified fungi perhaps indicate the loss of some known protective benefits of commensal fungi such as mannans, a key component of fungal cell wall. Jiang et al reported that mannans stimulation of mice intestine alone was sufficient to prevent disease susceptibility in mice depleted of commensal bacteria [45]. Composition of the gut microbiota is strongly involved in the maintenance and the shaping of the immune responses related to the intestinal barrier. Dysbiosis may lead to intestinal barrier disruption and increased susceptibility to certain diseases such as inflammatory bowel disease [46]. For example, a study found that *Candida albicans* mono-colonization efficiently reversed dextran sodium sulfate (DSS)-induced colitis after antibiotic induced eradication of commensal bacteria in mice [45]. Here, we reported a shift from the commensal *C*. *albicans into Candida glabrata* (% absolute −6.3%, p-value < 0.01) when infected with *H*. *pylori*. Interestingly and in contrast to *C*. *albicans*, West, Lara, et al. reported that inactivation of genes involved in mannan biosynthesis have been linked to increase virulence in *C*. *glabrata* [47]. However, whether this dissimilarity in mannan biosynthesis significantly alter intestinal barrier is yet to be elucidated.

We acknowledge potential limitations of this study, including relatively small sample size, more advanced functional analysis, and direct gastric biopsy to elucidate the disease pathogenesis and mucosal changes.

In conclusion, the role of *H*. *pylori* spans beyond gastric microbiota to possibly affects downstream gastrointestinal microbiota. Underscoring the importance of *H*. *pylori* associated changes in the gastrointestinal dysbiosis and its possible role in inflammation and colorectal carcinomas development. More studies connecting the gut microbiota-host-*H*. *pylori* interactions are needed to fully understand these associations and its effect on related illnesses.

## Supporting information

S1 Table Clinical characteristics. Metadata reflecting clinical characteristics including age, ethnicity, gender, and BMI of *H*. *pylori*-infected and uninfected subjects.
(DOCX)

S1 Fig Bacterial sequence curation and analysis. Sequenced 16S rRNA gene V4 amplicons generated from DNA samples on a MiSeq. MiSeq-generated Fastq files were quality-filtered and clustered into 97% similarity operational taxonomic units (OTUs) using the mothur software package [http://www.mothur.org]. The resulting dataset had 21257 OTUs (including those occurring once with a count of 1, or singletons). An average of 18383 quality-filtered reads were generated per sample. Sequencing quality for R1 and R2 was determined using FastQC 0.11.5, and visualized below.
(DOCX)

S2 Fig Fungal sequence curation and analysis. Sequenced ITS2 amplicons generated from DNA samples on a MiSeq. MiSeq-generated Fastq files were quality-filtered and clustered into 97% similarity operational taxonomic units (OTUs) using the mothur software package [http://www.mothur.org]. The resulting dataset had 3171 OTUs (including those occurring once with a count of 1, or singletons). An average of 9581 quality-filtered reads were generated per sample. Sequencing quality for R1 and R2 was determined using FastQC 0.11.5, and visualized below.
(DOCX)

S3 Fig Bacterial summary *taxonomic* composition. High quality reads classified using Greengenes v. 13_8 as the reference database. The aggregated OTUs into each taxonomic rank, and plotted the relative abundance of the most abundant ones. In the figure legends, the unfilled portion of the bar represents unclassified and lower-abundance taxa.
(DOCX)

S4 Fig Fungal summary *taxonomic* composition. High quality reads classified using Warcup V2 as the reference database. The aggregated OTUs into each taxonomic rank, and plotted the relative abundance of the most abundant ones. In the figure legends, the unfilled portion of the bar represents unclassified and lower-abundance taxa.
(DOCX)

## References

[1] Hooi JKY, Lai WY, Ng WK, Suen MMY, Underwood FE, Tanyingoh D, et al Global Prevalence of Helicobacter pylori Infection: Systematic Review and Meta-Analysis. Gastroenterology. 2017;153(2):420–9. Epub 2017/05/01. 10.1053/j.gastro.2017.04.022 28456631

[2] Cover TL, Blaser MJ. Helicobacter pylori in health and disease. Gastroenterology. 2009;136(6):1863–73. Epub 2009/05/22. 10.1053/j.gastro.2009.01.073 19457415 PMC3644425

[3] Sugizaki K, Tari A, Kitadai Y, Oda I, Nakamura S, Yoshino T, et al Anti-Helicobacter pylori therapy in localized gastric mucosa-associated lymphoid tissue lymphoma: A prospective, nationwide, multicenter study in Japan. Helicobacter. 2018;23(2):e12474 Epub 2018/03/06. 10.1111/hel.12474 29504247 PMC5900897

[4] Butt J, Varga MG, Blot WJ, Teras L, Visvanathan K, Le Marchand L, et al Serologic Response to Helicobacter pylori Proteins Associated With Risk of Colorectal Cancer Among Diverse Populations in the United States. Gastroenterology. 2019;156(1):175–86 e2. Epub 2018/10/09. 10.1053/j.gastro.2018.09.054 30296434 PMC6309494

[5] Park H, Park JJ, Park YM, Baik SJ, Lee HJ, Jung DH, et al The association between Helicobacter pylori infection and the risk of advanced colorectal neoplasia may differ according to age and cigarette smoking. Helicobacter. 2018;23(3):e12477 Epub 2018/03/31. 10.1111/hel.12477 29600573

[6] Hatakeyama M, Brzozowski T. Pathogenesis of Helicobacter pylori infection. Helicobacter. 2006;11 Suppl 1:14–20. Epub 2006/08/24. 16925606 10.1111/j.1478-405X.2006.00424.x

[7] Bravo D, Hoare A, Soto C, Valenzuela MA, Quest AF. Helicobacter pylori in human health and disease: Mechanisms for local gastric and systemic effects. World journal of gastroenterology. 2018;24(28):3071–89. Epub 2018/08/02. 10.3748/wjg.v24.i28.3071 30065554 PMC6064966

[8] Sheh A, Fox JG. The role of the gastrointestinal microbiome in Helicobacter pylori pathogenesis. Gut microbes. 2013;4(6):505–31. Epub 2013/08/22. 10.4161/gmic.26205 23962822 PMC3928162

[9] Schulz C, Schutte K, Koch N, Vilchez-Vargas R, Wos-Oxley ML, Oxley APA, et al The active bacterial assemblages of the upper GI tract in individuals with and without Helicobacter infection. Gut. 2018;67(2):216–25. Epub 2016/12/07. 10.1136/gutjnl-2016-312904 27920199

[10] Coker OO, Dai Z, Nie Y, Zhao G, Cao L, Nakatsu G, et al Mucosal microbiome dysbiosis in gastric carcinogenesis. Gut. 2018;67(6):1024–32. Epub 2017/08/03. 10.1136/gutjnl-2017-314281 28765474 PMC5969346

[11] Ferreira RM, Pereira-Marques J, Pinto-Ribeiro I, Costa JL, Carneiro F, Machado JC, et al Gastric microbial community profiling reveals a dysbiotic cancer-associated microbiota. Gut. 2018;67(2):226–36. Epub 2017/11/06. 10.1136/gutjnl-2017-314205 29102920 PMC5868293

[12] Llorca L, Perez-Perez G, Urruzuno P, Martinez MJ, Iizumi T, Gao Z, et al Characterization of the Gastric Microbiota in a Pediatric Population According to Helicobacter pylori Status. The Pediatric infectious disease journal. 2017;36(2):173–8. Epub 2016/11/08. 10.1097/INF.0000000000001383 27820723

[13] Benavides-Ward A, Vasquez-Achaya F, Silva-Caso W, Aguilar-Luis MA, Mazulis F, Urteaga N, et al Helicobacter pylori and its relationship with variations of gut microbiota in asymptomatic children between 6 and 12 years. BMC research notes. 2018;11(1):468 Epub 2018/07/15. 10.1186/s13104-018-3565-5 30005690 PMC6043948

[14] Kozich JJ, Westcott SL, Baxter NT, Highlander SK, Schloss PD. Development of a dual-index sequencing strategy and curation pipeline for analyzing amplicon sequence data on the MiSeq Illumina sequencing platform. Applied and environmental microbiology. 2013;79(17):5112–20. Epub 2013/06/25. 10.1128/AEM.01043-13 23793624 PMC3753973

[15] Schloss PD, Westcott SL, Ryabin T, Hall JR, Hartmann M, Hollister EB, et al Introducing mothur: open-source, platform-independent, community-supported software for describing and comparing microbial communities. Applied and environmental microbiology. 2009;75(23):7537–41. Epub 2009/10/06. 10.1128/AEM.01541-09 19801464 PMC2786419

[16] Gweon HS, Oliver A, Taylor J, Booth T, Gibbs M, Read DS, et al PIPITS: an automated pipeline for analyses of fungal internal transcribed spacer sequences from the Illumina sequencing platform. Methods in ecology and evolution. 2015;6(8):973–80. Epub 2016/08/30. 10.1111/2041-210X.12399 27570615 PMC4981123

[17] Maldonado-Contreras A, Goldfarb KC, Godoy-Vitorino F, Karaoz U, Contreras M, Blaser MJ, et al Structure of the human gastric bacterial community in relation to Helicobacter pylori status. The ISME journal. 2011;5(4):574–9. Epub 2010/10/12. 10.1038/ismej.2010.149 20927139 PMC3105737

[18] Chen L, Xu W, Lee A, He J, Huang B, Zheng W, et al The impact of Helicobacter pylori infection, eradication therapy and probiotic supplementation on gut microenvironment homeostasis: An open-label, randomized clinical trial. EBioMedicine. 2018;35:87–96. Epub 2018/08/27. 10.1016/j.ebiom.2018.08.028 30145102 PMC6161473

[19] Espinoza JL, Matsumoto A, Tanaka H, Matsumura I. Gastric microbiota: An emerging player in Helicobacter pylori-induced gastric malignancies. Cancer letters. 2018;414:147–52. Epub 2017/11/16. 10.1016/j.canlet.2017.11.009 29138097

[20] Zhao Y, Gao X, Guo J, Yu D, Xiao Y, Wang H, et al Helicobacter pylori infection alters gastric and tongue coating microbial communities. Helicobacter. 2019;24(2):e12567 Epub 2019/02/09. 10.1111/hel.12567 30734438 PMC6593728

[21] Gantuya B, El-Serag HB, Matsumoto T, Ajami NJ, Oyuntsetseg K, Azzaya D, et al Gastric Microbiota in Helicobacter pylori-Negative and -Positive Gastritis Among High Incidence of Gastric Cancer Area. Cancers. 2019;11(4). Epub 2019/04/13. 10.3390/cancers11040504 PMC6520852 30974798

[22] Yatsunenko T, Rey FE, Manary MJ, Trehan I, Dominguez-Bello MG, Contreras M, et al Human gut microbiome viewed across age and geography. Nature. 2012;486(7402):222–7. Epub 2012/06/16. 10.1038/nature11053 22699611 PMC3376388

[23] Ley RE, Turnbaugh PJ, Klein S, Gordon JI. Microbial ecology: human gut microbes associated with obesity. Nature. 2006;444(7122):1022–3. Epub 2006/12/22. 10.1038/4441022a 17183309

[24] Turnbaugh PJ, Backhed F, Fulton L, Gordon JI. Diet-induced obesity is linked to marked but reversible alterations in the mouse distal gut microbiome. Cell host & microbe. 2008;3(4):213–23. Epub 2008/04/15. 18407065 10.1016/j.chom.2008.02.015 PMC3687783

[25] Lozupone CA, Stombaugh JI, Gordon JI, Jansson JK, Knight R. Diversity, stability and resilience of the human gut microbiota. Nature. 2012;489(7415):220–30. Epub 2012/09/14. 10.1038/nature11550 22972295 PMC3577372

[26] Oh B, Kim JW, Kim BS. Changes in the Functional Potential of the Gut Microbiome Following Probiotic Supplementation during Helicobacter Pylori Treatment. Helicobacter. 2016;21(6):493–503. Epub 2016/03/19. 10.1111/hel.12306 26991862

[27] Queiroz DM, Rocha AM, Crabtree JE. Unintended consequences of Helicobacter pylori infection in children in developing countries: iron deficiency, diarrhea, and growth retardation. Gut microbes. 2013;4(6):494–504. Epub 2013/08/31. 10.4161/gmic.26277 23988829 PMC3928161

[28] Hudak L, Jaraisy A, Haj S, Muhsen K. An updated systematic review and meta-analysis on the association between Helicobacter pylori infection and iron deficiency anemia. Helicobacter. 2017;22(1). Epub 2016/07/14. 10.1111/hel.12330 27411077

[29] Thomson MJ, Pritchard DM, Boxall SA, Abuderman AA, Williams JM, Varro A, et al Gastric Helicobacter infection induces iron deficiency in the INS-GAS mouse. PloS one. 2012;7(11):e50194 Epub 2012/11/28. 10.1371/journal.pone.0050194 23185574 PMC3501456

[30] Noto JM, Gaddy JA, Lee JY, Piazuelo MB, Friedman DB, Colvin DC, et al Iron deficiency accelerates Helicobacter pylori-induced carcinogenesis in rodents and humans. The Journal of clinical investigation. 2013;123(1):479–92. Epub 2012/12/22. 10.1172/JCI64373 23257361 PMC3533289

[31] Werner T, Wagner SJ, Martinez I, Walter J, Chang JS, Clavel T, et al Depletion of luminal iron alters the gut microbiota and prevents Crohn's disease-like ileitis. Gut. 2011;60(3):325–33. Epub 2010/11/16. 10.1136/gut.2010.216929 21076126

[32] Marchesi JR, Dutilh BE, Hall N, Peters WH, Roelofs R, Boleij A, et al Towards the human colorectal cancer microbiome. PloS one. 2011;6(5):e20447 Epub 2011/06/08. 10.1371/journal.pone.0020447 21647227 PMC3101260

[33] Burns MB, Montassier E, Abrahante J, Priya S, Niccum DE, Khoruts A, et al Colorectal cancer mutational profiles correlate with defined microbial communities in the tumor microenvironment. PLoS genetics. 2018;14(6):e1007376 Epub 2018/06/21. 10.1371/journal.pgen.1007376 29924794 PMC6028121

[34] Tjalsma H, Boleij A, Marchesi JR, Dutilh BE. A bacterial driver-passenger model for colorectal cancer: beyond the usual suspects. Nature reviews Microbiology. 2012;10(8):575–82. Epub 2012/06/26. 10.1038/nrmicro2819 22728587

[35] Kostic AD, Chun E, Robertson L, Glickman JN, Gallini CA, Michaud M, et al Fusobacterium nucleatum potentiates intestinal tumorigenesis and modulates the tumor-immune microenvironment. Cell host & microbe. 2013;14(2):207–15. Epub 2013/08/21. 23954159 10.1016/j.chom.2013.07.007 PMC3772512

[36] Farhana L, Banerjee HN, Verma M, Majumdar APN. Role of Microbiome in Carcinogenesis Process and Epigenetic Regulation of Colorectal Cancer. Methods Mol Biol. 2018;1856:35–55. Epub 2018/09/05. 10.1007/978-1-4939-8751-1_3 30178245

[37] Herstad KMV, Moen AEF, Gaby JC, Moe L, Skancke E. Characterization of the fecal and mucosa-associated microbiota in dogs with colorectal epithelial tumors. PloS one. 2018;13(5):e0198342 Epub 2018/06/01. 10.1371/journal.pone.0198342 29852000 PMC5979030

[38] Strofilas A, Lagoudianakis EE, Seretis C, Pappas A, Koronakis N, Keramidaris D, et al Association of helicobacter pylori infection and colon cancer. Journal of clinical medicine research. 2012;4(3):172–6. Epub 2012/06/22. 10.4021/jocmr880w 22719803 PMC3376875

[39] Gorbach SL, Plaut AG, Nahas L, Weinstein L, Spanknebel G, Levitan R. Studies of intestinal microflora. II. Microorganisms of the small intestine and their relations to oral and fecal flora. Gastroenterology. 1967;53(6):856–67. Epub 1967/12/01. 4863722

[40] Wheeler ML, Limon JJ, Bar AS, Leal CA, Gargus M, Tang J, et al Immunological Consequences of Intestinal Fungal Dysbiosis. Cell host & microbe. 2016;19(6):865–73. Epub 2016/05/31. 27237365 10.1016/j.chom.2016.05.003 PMC4900921

[41] Sokol H, Leducq V, Aschard H, Pham HP, Jegou S, Landman C, et al Fungal microbiota dysbiosis in IBD. Gut. 2017;66(6):1039–48. Epub 2016/02/05. 10.1136/gutjnl-2015-310746 26843508 PMC5532459

[42] Zuo T, Wong SH, Cheung CP, Lam K, Lui R, Cheung K, et al Gut fungal dysbiosis correlates with reduced efficacy of fecal microbiota transplantation in Clostridium difficile infection. Nature communications. 2018;9(1):3663 Epub 2018/09/12. 10.1038/s41467-018-06103-6 30202057 PMC6131390

[43] Limon JJ, Skalski JH, Underhill DM. Commensal Fungi in Health and Disease. Cell host & microbe. 2017;22(2):156–65. Epub 2017/08/12. 28799901 10.1016/j.chom.2017.07.002 PMC5573128

[44] Hoffmann C, Dollive S, Grunberg S, Chen J, Li H, Wu GD, et al Archaea and fungi of the human gut microbiome: correlations with diet and bacterial residents. PloS one. 2013;8(6):e66019 Epub 2013/06/27. 10.1371/journal.pone.0066019 23799070 PMC3684604

[45] Jiang TT, Shao TY, Ang WXG, Kinder JM, Turner LH, Pham G, et al Commensal Fungi Recapitulate the Protective Benefits of Intestinal Bacteria. Cell host & microbe. 2017;22(6):809–16 e4. Epub 2017/11/28. 29174402 10.1016/j.chom.2017.10.013 PMC5730478

[46] Hamilton MK, Boudry G, Lemay DG, Raybould HE. Changes in intestinal barrier function and gut microbiota in high-fat diet-fed rats are dynamic and region dependent. American journal of physiology Gastrointestinal and liver physiology. 2015;308(10):G840–51. Epub 2015/03/10. 10.1152/ajpgi.00029.2015 25747351 PMC4437018

[47] West L, Lowman DW, Mora-Montes HM, Grubb S, Murdoch C, Thornhill MH, et al Differential virulence of Candida glabrata glycosylation mutants. The Journal of biological chemistry. 2013;288(30):22006–18. Epub 2013/05/31. 10.1074/jbc.M113.478743 23720756 PMC3724654

