# Correction: Exploring the impact of *Helicobacter pylori* on gut microbiome composition

**DOI:** 10.1371/journal.pone.0256274

**Published:** 2021-08-11

**Authors:** Nihar Ranjan Dash, Ghalia Khoder, Aml Mohamed Nada, Mohammad Tahseen Al Bataineh

An additional affiliation is missing for the third author. Aml Mohamed Nada is also affiliated with Internal Medicine, Mansoura University, Mansoura, Egypt.
