# Supplementary material for: Exploring the impact of Helicobacter pylori on gut microbiome composition
Source: PLoS One. 2019 Jun 18;14(6):e0218274. doi: 10.1371/journal.pone.0218274 (PMC6581275; doi:10.1371/journal.pone.0218274)
Supplement: S1 Table — Metadata reflecting clinical characteristics including age, ethnicity, gender, and BMI of H. pylori-infected and uninfected subjects. (DOCX) [file pone.0218274.s001.docx]

**S1 Table. Clinical characteristics.**

Metadata reflecting clinical characteristics including age, ethnicity, gender, and BMI of *H. pylori*-infected and uninfected subjects

|  | *H. pylori*-infected subjects **(n = 12)** | uninfected subjects **(n = 48)** |
| --- | --- | --- |
| **Age, years**  **Mean (SD, range)** | **47.3 (15.5, 49)** | **45.9 (20.1, 59)** |
| **Ethnicity,**  **Emirati (%)** | **100%** | **100%** |
| **Gender (M%, F%)** | **(25, 75)** | **(25, 75)** |
| **BMI(Kg/m^2^) Mean (SD, range)** | **32.6 (7.3, 28.1)** | **27.3 (5.6, 20.8)** |
