# Supplementary material for: Exploring the impact of Helicobacter pylori on gut microbiome composition
Source: PLoS One. 2019 Jun 18;14(6):e0218274. doi: 10.1371/journal.pone.0218274 (PMC6581275; doi:10.1371/journal.pone.0218274)

**S1 Fig. Bacterial sequence curation and analysis**

Sequenced 16S rRNA gene V4 amplicons generated from DNA samples on a MiSeq. MiSeq-generated Fastq files were quality-filtered and clustered into 97% similarity operational taxonomic units (OTUs) using the mothur software package [[http://www.mothur.org](http://www.mothur.org/)]. The resulting dataset had 21257 OTUs (including those occurring once with a count of 1, or singletons). An average of 18383 quality-filtered reads were generated per sample. Sequencing quality for R1 and R2 was determined using FastQC 0.11.5, and visualized below.


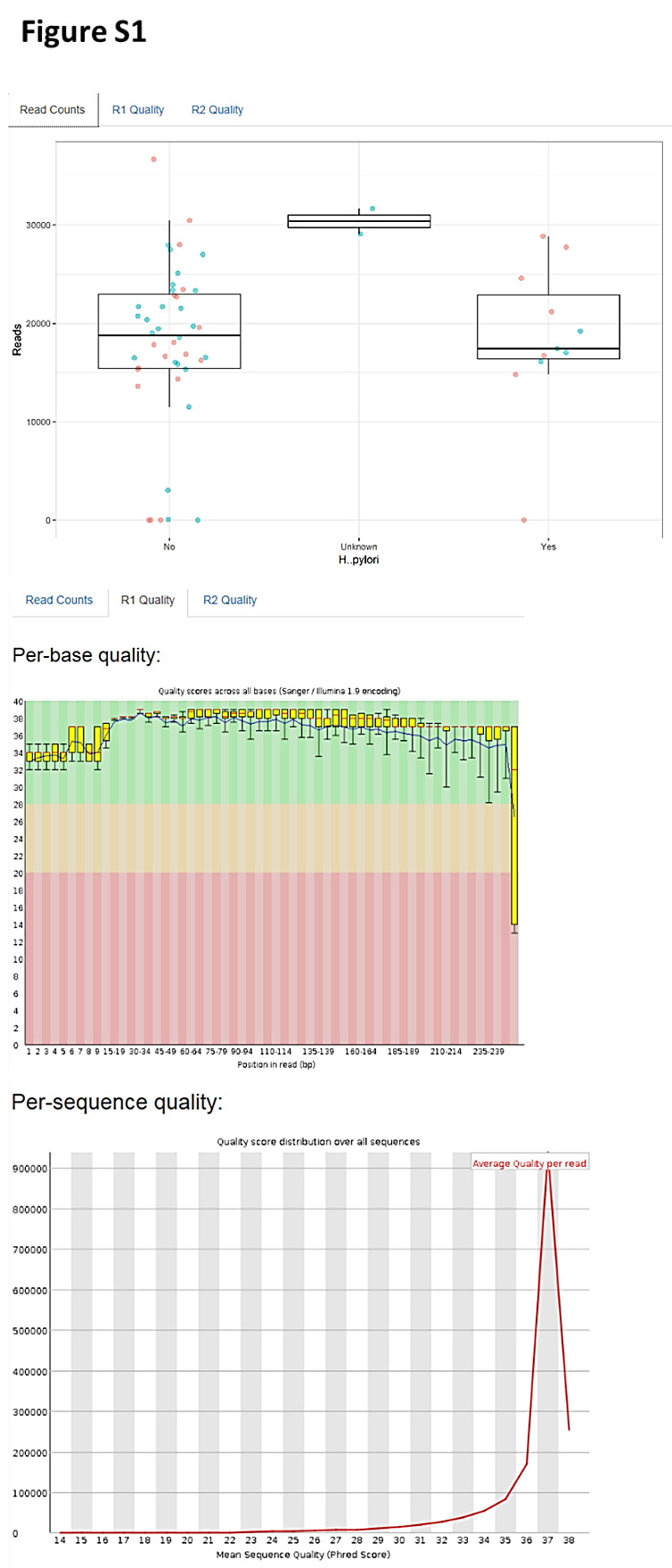

Supplement: S1 Fig — Sequenced 16S rRNA gene V4 amplicons generated from DNA samples on a MiSeq. MiSeq-generated Fastq files were quality-filtered and clustered into 97% similarity operational taxonomic units (OTUs) using the mothur software package [http://www.mothur.org]. The resulting dataset had 21257 OTUs (including those occurring once with a count of 1, or singletons). An average of 18383 quality-filtered reads were generated per sample. Sequencing quality for R1 and R2 was determined using FastQC 0.11.5, and visualized below. (DOCX) [file pone.0218274.s002.docx]
