# Supplementary material for: Exploring the impact of Helicobacter pylori on gut microbiome composition
Source: PLoS One. 2019 Jun 18;14(6):e0218274. doi: 10.1371/journal.pone.0218274 (PMC6581275; doi:10.1371/journal.pone.0218274)

# S3 Fig. Bacterial summary taxonomic composition

High quality reads classified using Greengenes v. 13_8 as the reference database. The aggregated OTUs into each taxonomic rank, and plotted the relative abundance of the most abundant ones. In the figure legends, the unfilled portion of the bar represents unclassified and lower-abundance taxa.


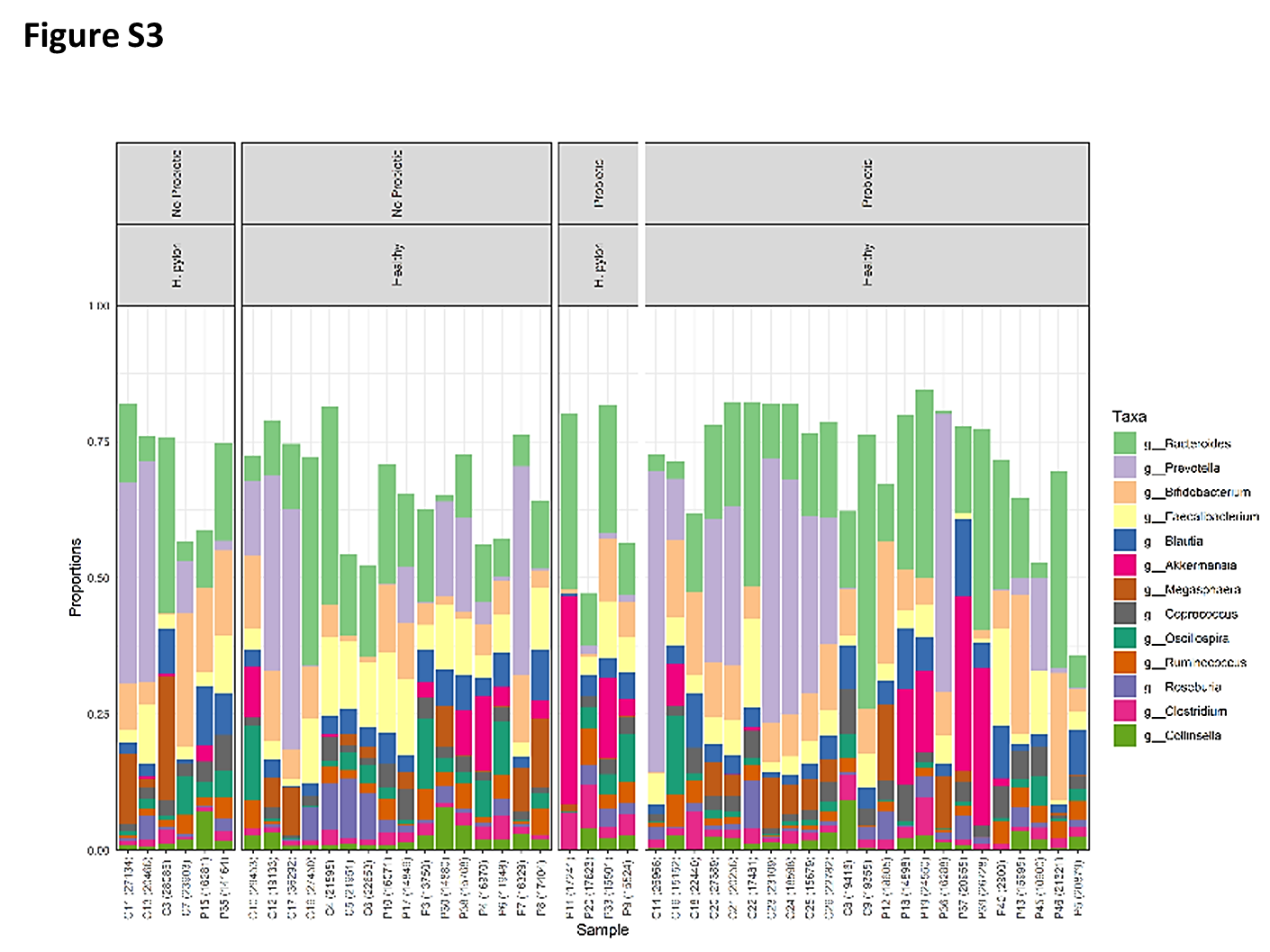

Supplement: S3 Fig — High quality reads classified using Greengenes v. 13_8 as the reference database. The aggregated OTUs into each taxonomic rank, and plotted the relative abundance of the most abundant ones. In the figure legends, the unfilled portion of the bar represents unclassified and lower-abundance taxa. (DOCX) [file pone.0218274.s004.docx]
